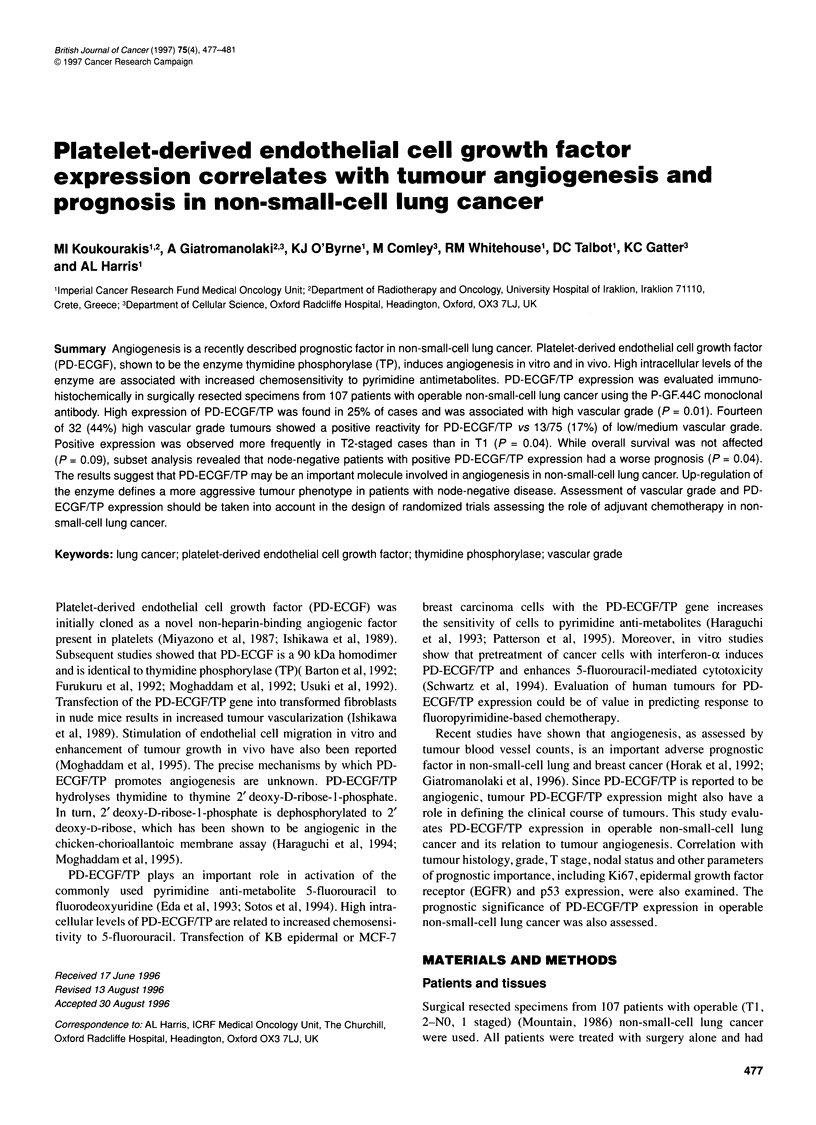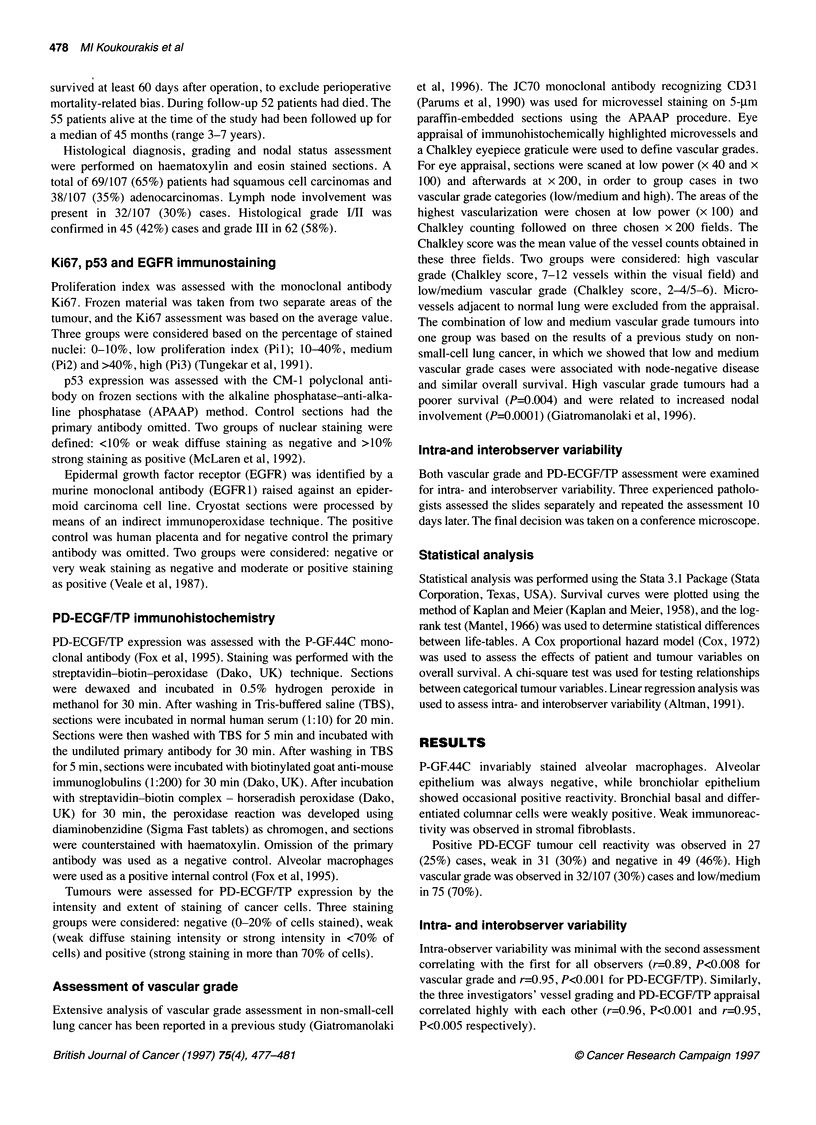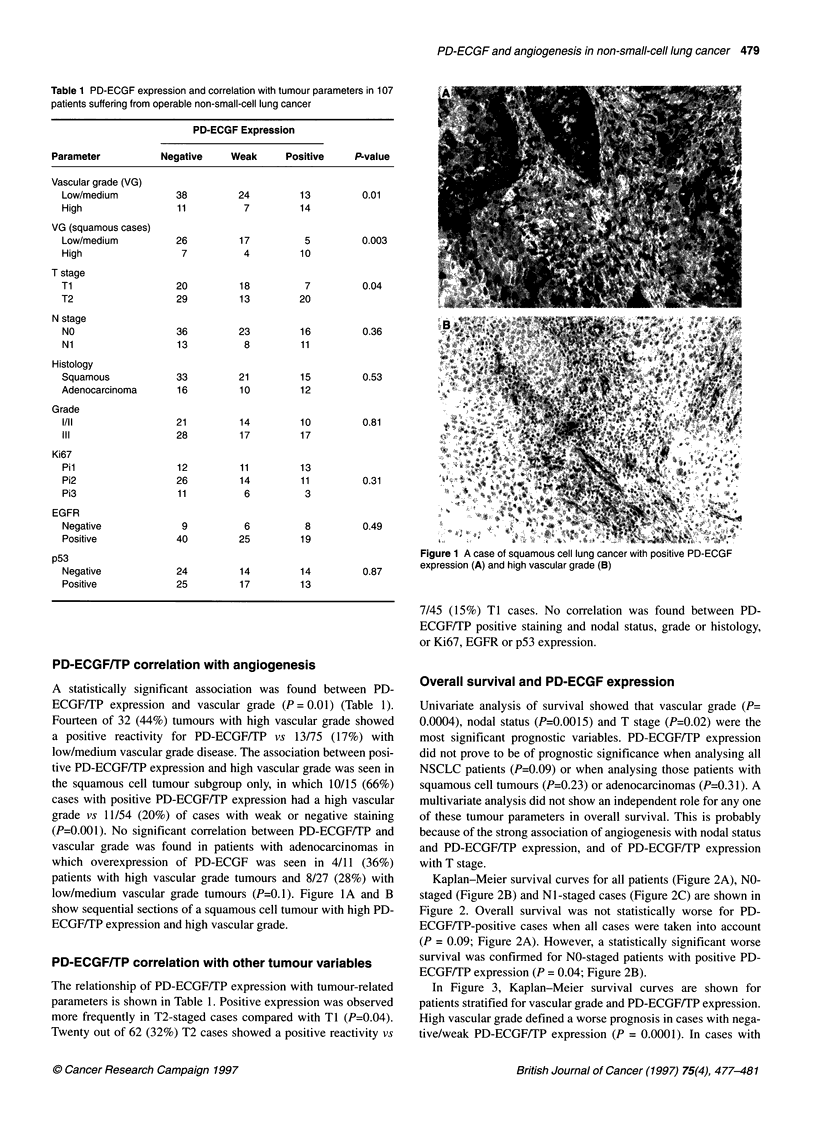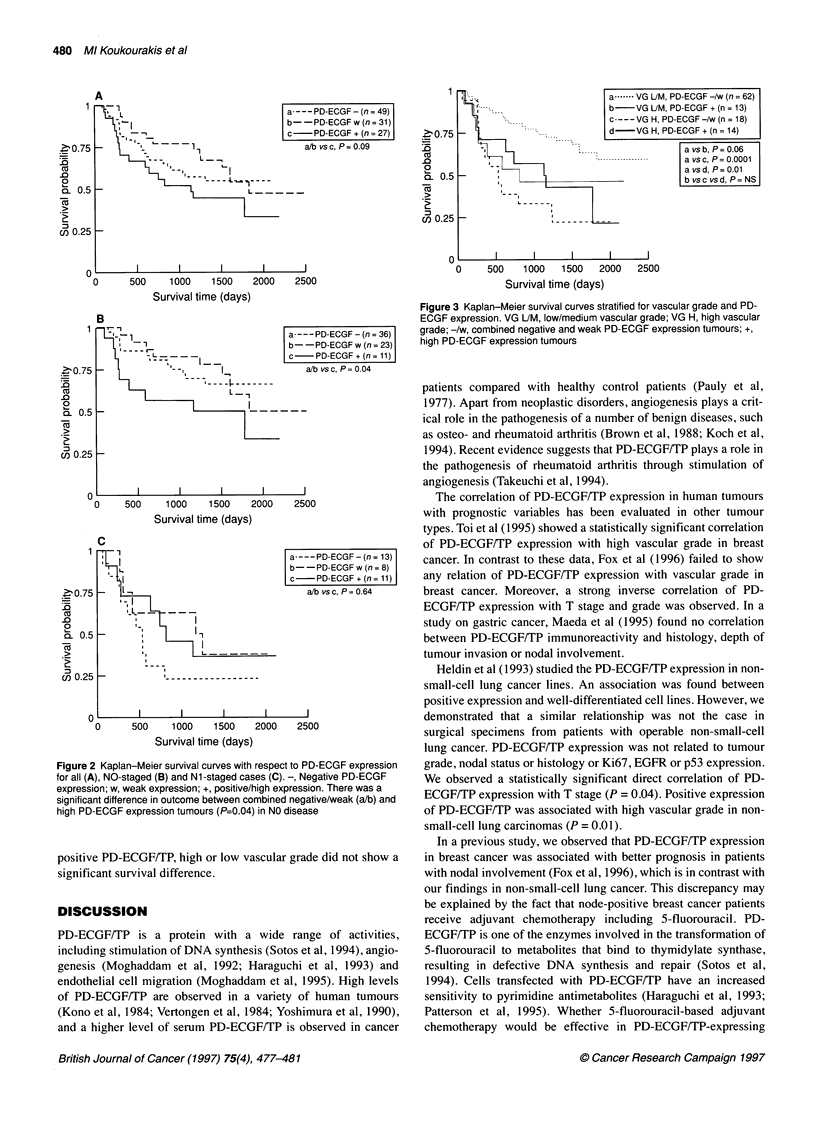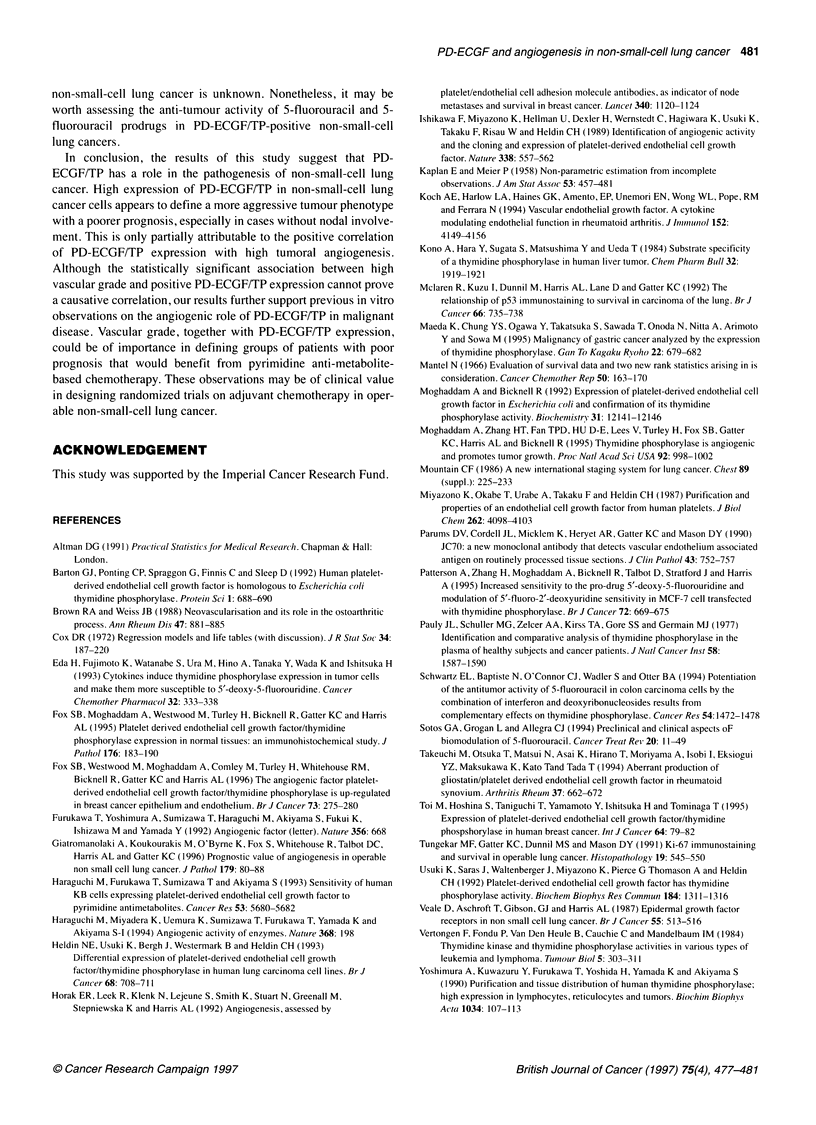# Platelet-derived endothelial cell growth factor expression correlates with tumour angiogenesis and prognosis in non-small-cell lung cancer.

**DOI:** 10.1038/bjc.1997.83

**Published:** 1997

**Authors:** M. I. Koukourakis, A. Giatromanolaki, K. J. O'Byrne, M. Comley, R. M. Whitehouse, D. C. Talbot, K. C. Gatter, A. L. Harris

**Affiliations:** Department of Radiotherapy and Oncology, University Hospital of Iraklion, Crete, Greece.

## Abstract

**Images:**


British Journal of Cancer (1997) 75(4), 477–481
© 1997 Cancer Research Campaign

### Platelet-derived endothelial cell growth factor expression correlates with tumour angiogenesis and prognosis in non-small-cell lung cancer

**MI Koukourakis[1,2], A Giatromanolaki[2,3], KJ O'Byrne[1], M Comley[3], RM Whitehouse[1], DC Talbot[1], KC Gatter[3] and AL Harris[1]**

[1]Imperial Cancer Research Fund Medical Oncology Unit; [2]Department of Radiotherapy and Oncology, University Hospital of Iraklion, Iraklion 71110, Crete, Greece; [3]Department of Cellular Science, Oxford Radcliffe Hospital, Headington, Oxford, OX3 7LJ, UK

**Summary** Angiogenesis is a recently described prognostic factor in non-small-cell lung cancer. Platelet-derived endothelial cell growth factor (PD-ECGF), shown to be the enzyme thymidine phosphorylase (TP), induces angiogenesis in vitro and in vivo. High intracellular levels of the enzyme are associated with increased chemosensitivity to pyrimidine antimetabolites. PD-ECGF/TP expression was evaluated immunohistochemically in surgically resected specimens from 107 patients with operable non-small-cell lung cancer using the P-GF.44C monoclonal antibody. High expression of PD-ECGF/TP was found in 25% of cases and was associated with high vascular grade ($P$ = 0.01). Fourteen of 32 (44%) high vascular grade tumours showed a positive reactivity for PD-ECGF/TP *vs* 13/75 (17%) of low/medium vascular grade. Positive expression was observed more frequently in T2-staged cases than in T1 ($P$ = 0.04). While overall survival was not affected ($P$ = 0.09), subset analysis revealed that node-negative patients with positive PD-ECGF/TP expression had a worse prognosis ($P$ = 0.04). The results suggest that PD-ECGF/TP may be an important molecule involved in angiogenesis in non-small-cell lung cancer. Up-regulation of the enzyme defines a more aggressive tumour phenotype in patients with node-negative disease. Assessment of vascular grade and PD-ECGF/TP expression should be taken into account in the design of randomized trials assessing the role of adjuvant chemotherapy in non-small-cell lung cancer.

**Keywords:** lung cancer; platelet-derived endothelial cell growth factor; thymidine phosphorylase; vascular grade

Platelet-derived endothelial cell growth factor (PD-ECGF) was initially cloned as a novel non-heparin-binding angiogenic factor present in platelets (Miyazono et al, 1987; Ishikawa et al, 1989). Subsequent studies showed that PD-ECGF is a 90 kDa homodimer and is identical to thymidine phosphorylase (TP)( Barton et al, 1992; Furukuru et al, 1992; Moghaddam et al, 1992; Usuki et al, 1992). Transfection of the PD-ECGF/TP gene into transformed fibroblasts in nude mice results in increased tumour vascularization (Ishikawa et al, 1989). Stimulation of endothelial cell migration in vitro and enhancement of tumour growth in vivo have also been reported (Moghaddam et al, 1995). The precise mechanisms by which PD-ECGF/TP promotes angiogenesis are unknown. PD-ECGF/TP hydrolyses thymidine to thymine 2′ deoxy-D-ribose-1-phosphate. In turn, 2′ deoxy-D-ribose-1-phosphate is dephosphorylated to 2′ deoxy-D-ribose, which has been shown to be angiogenic in the chicken-chorioallantoic membrane assay (Haraguchi et al, 1994; Moghaddam et al, 1995).

PD-ECGF/TP plays an important role in activation of the commonly used pyrimidine anti-metabolite 5-fluorouracil to fluorodeoxyuridine (Eda et al, 1993; Sotos et al, 1994). High intracellular levels of PD-ECGF/TP are related to increased chemosensitivity to 5-fluorouracil. Transfection of KB epidermal or MCF-7 breast carcinoma cells with the PD-ECGF/TP gene increases the sensitivity of cells to pyrimidine anti-metabolites (Haraguchi et al, 1993; Patterson et al, 1995). Moreover, in vitro studies show that pretreatment of cancer cells with interferon-α induces PD-ECGF/TP and enhances 5-fluorouracil-mediated cytotoxicity (Schwartz et al, 1994). Evaluation of human tumours for PD-ECGF/TP expression could be of value in predicting response to fluoropyrimidine-based chemotherapy.

Recent studies have shown that angiogenesis, as assessed by tumour blood vessel counts, is an important adverse prognostic factor in non-small-cell lung and breast cancer (Horak et al, 1992; Giatromanolaki et al, 1996). Since PD-ECGF/TP is reported to be angiogenic, tumour PD-ECGF/TP expression might also have a role in defining the clinical course of tumours. This study evaluates PD-ECGF/TP expression in operable non-small-cell lung cancer and its relation to tumour angiogenesis. Correlation with tumour histology, grade, T stage, nodal status and other parameters of prognostic importance, including Ki67, epidermal growth factor receptor (EGFR) and p53 expression, were also examined. The prognostic significance of PD-ECGF/TP expression in operable non-small-cell lung cancer was also assessed.

### MATERIALS AND METHODS

### Patients and tissues

Surgical resected specimens from 107 patients with operable (T1, 2–N0, 1 staged) (Mountain, 1986) non-small-cell lung cancer were used. All patients were treated with surgery alone and had

*Received 17 June 1996*
*Revised 13 August 1996*
*Accepted 30 August 1996*

*Correspondence to:* AL Harris, ICRF Medical Oncology Unit, The Churchill, Oxford Radcliffe Hospital, Headington, Oxford OX3 7LJ, UK

survived at least 60 days after operation, to exclude perioperative mortality-related bias. During follow-up 52 patients had died. The 55 patients alive at the time of the study had been followed up for a median of 45 months (range 3–7 years).

Histological diagnosis, grading and nodal status assessment were performed on haematoxylin and eosin stained sections. A total of 69/107 (65%) patients had squamous cell carcinomas and 38/107 (35%) adenocarcinomas. Lymph node involvement was present in 32/107 (30%) cases. Histological grade I/II was confirmed in 45 (42%) cases and grade III in 62 (58%).

### Ki67, p53 and EGFR immunostaining

Proliferation index was assessed with the monoclonal antibody Ki67. Frozen material was taken from two separate areas of the tumour, and the Ki67 assessment was based on the average value. Three groups were considered based on the percentage of stained nuclei: 0–10%, low proliferation index (Pi1); 10–40%, medium (Pi2) and >40%, high (Pi3) (Tungekar et al, 1991).

p53 expression was assessed with the CM-1 polyclonal antibody on frozen sections with the alkaline phosphatase–anti-alkaline phosphatase (APAAP) method. Control sections had the primary antibody omitted. Two groups of nuclear staining were defined: <10% or weak diffuse staining as negative and >10% strong staining as positive (McLaren et al, 1992).

Epidermal growth factor receptor (EGFR) was identified by a murine monoclonal antibody (EGFR1) raised against an epidermoid carcinoma cell line. Cryostat sections were processed by means of an indirect immunoperoxidase technique. The positive control was human placenta and for negative control the primary antibody was omitted. Two groups were considered: negative or very weak staining as negative and moderate or positive staining as positive (Veale et al, 1987).

### PD-ECGF/TP immunohistochemistry

PD-ECGF/TP expression was assessed with the P-GF.44C monoclonal antibody (Fox et al, 1995). Staining was performed with the streptavidin–biotin–peroxidase (Dako, UK) technique. Sections were dewaxed and incubated in 0.5% hydrogen peroxide in methanol for 30 min. After washing in Tris-buffered saline (TBS), sections were incubated in normal human serum (1:10) for 20 min. Sections were then washed with TBS for 5 min and incubated with the undiluted primary antibody for 30 min. After washing in TBS for 5 min, sections were incubated with biotinylated goat anti-mouse immunoglobulins (1:200) for 30 min (Dako, UK). After incubation with streptavidin–biotin complex – horseradish peroxidase (Dako, UK) for 30 min, the peroxidase reaction was developed using diaminobenzidine (Sigma Fast tablets) as chromogen, and sections were counterstained with haematoxylin. Omission of the primary antibody was used as a negative control. Alveolar macrophages were used as a positive internal control (Fox et al, 1995).

Tumours were assessed for PD-ECGF/TP expression by the intensity and extent of staining of cancer cells. Three staining groups were considered: negative (0–20% of cells stained), weak (weak diffuse staining intensity or strong intensity in <70% of cells) and positive (strong staining in more than 70% of cells).

### Assessment of vascular grade

Extensive analysis of vascular grade assessment in non-small-cell lung cancer has been reported in a previous study (Giatromanolaki et al, 1996). The JC70 monoclonal antibody recognizing CD31 (Parums et al, 1990) was used for microvessel staining on 5-µm paraffin-embedded sections using the APAAP procedure. Eye appraisal of immunohistochemically highlighted microvessels and a Chalkley eyepiece graticule were used to define vascular grades. For eye appraisal, sections were scaned at low power (× 40 and × 100) and afterwards at × 200, in order to group cases in two vascular grade categories (low/medium and high). The areas of the highest vascularization were chosen at low power (× 100) and Chalkley counting followed on three chosen × 200 fields. The Chalkley score was the mean value of the vessel counts obtained in these three fields. Two groups were considered: high vascular grade (Chalkley score, 7–12 vessels within the visual field) and low/medium vascular grade (Chalkley score, 2–4/5–6). Microvessels adjacent to normal lung were excluded from the appraisal. The combination of low and medium vascular grade tumours into one group was based on the results of a previous study on non-small-cell lung cancer, in which we showed that low and medium vascular grade cases were associated with node-negative disease and similar overall survival. High vascular grade tumours had a poorer survival ($P$=0.004) and were related to increased nodal involvement ($P$=0.0001) (Giatromanolaki et al, 1996).

### Intra-and interobserver variability

Both vascular grade and PD-ECGF/TP assessment were examined for intra- and interobserver variability. Three experienced pathologists assessed the slides separately and repeated the assessment 10 days later. The final decision was taken on a conference microscope.

### Statistical analysis

Statistical analysis was performed using the Stata 3.1 Package (Stata Corporation, Texas, USA). Survival curves were plotted using the method of Kaplan and Meier (Kaplan and Meier, 1958), and the log-rank test (Mantel, 1966) was used to determine statistical differences between life-tables. A Cox proportional hazard model (Cox, 1972) was used to assess the effects of patient and tumour variables on overall survival. A chi-square test was used for testing relationships between categorical tumour variables. Linear regression analysis was used to assess intra- and interobserver variability (Altman, 1991).

### RESULTS

P-GF.44C invariably stained alveolar macrophages. Alveolar epithelium was always negative, while bronchiolar epithelium showed occasional positive reactivity. Bronchial basal and differentiated columnar cells were weakly positive. Weak immunoreactivity was observed in stromal fibroblasts.

Positive PD-ECGF tumour cell reactivity was observed in 27 (25%) cases, weak in 31 (30%) and negative in 49 (46%). High vascular grade was observed in 32/107 (30%) cases and low/medium in 75 (70%).

### Intra- and interobserver variability

Intra-observer variability was minimal with the second assessment correlating with the first for all observers ($r$=0.89, $P$<0.008 for vascular grade and $r$=0.95, $P$<0.001 for PD-ECGF/TP). Similarly, the three investigators' vessel grading and PD-ECGF/TP appraisal correlated highly with each other ($r$=0.96, P<0.001 and $r$=0.95, P<0.005 respectively).

© Cancer Research Campaign 1997

**Table 1** PD-ECGF expression and correlation with tumour parameters in 107 patients suffering from operable non-small-cell lung cancer

| Parameter | PD-ECGF Expression | | | *P*-value |
|---|---|---|---|---|
| | Negative | Weak | Positive | |
| Vascular grade (VG) | | | | |
| Low/medium | 38 | 24 | 13 | 0.01 |
| High | 11 | 7 | 14 | |
| VG (squamous cases) | | | | |
| Low/medium | 26 | 17 | 5 | 0.003 |
| High | 7 | 4 | 10 | |
| T stage | | | | |
| T1 | 20 | 18 | 7 | 0.04 |
| T2 | 29 | 13 | 20 | |
| N stage | | | | |
| N0 | 36 | 23 | 16 | 0.36 |
| N1 | 13 | 8 | 11 | |
| Histology | | | | |
| Squamous | 33 | 21 | 15 | 0.53 |
| Adenocarcinoma | 16 | 10 | 12 | |
| Grade | | | | |
| I/II | 21 | 14 | 10 | 0.81 |
| III | 28 | 17 | 17 | |
| Ki67 | | | | |
| Pi1 | 12 | 11 | 13 | |
| Pi2 | 26 | 14 | 11 | 0.31 |
| Pi3 | 11 | 6 | 3 | |
| EGFR | | | | |
| Negative | 9 | 6 | 8 | 0.49 |
| Positive | 40 | 25 | 19 | |
| p53 | | | | |
| Negative | 24 | 14 | 14 | 0.87 |
| Positive | 25 | 17 | 13 | |

### PD-ECGF/TP correlation with angiogenesis

A statistically significant association was found between PD-ECGF/TP expression and vascular grade ($P = 0.01$) (Table 1). Fourteen of 32 (44%) tumours with high vascular grade showed a positive reactivity for PD-ECGF/TP *vs* 13/75 (17%) with low/medium vascular grade disease. The association between positive PD-ECGF/TP expression and high vascular grade was seen in the squamous cell tumour subgroup only, in which 10/15 (66%) cases with positive PD-ECGF/TP expression had a high vascular grade *vs* 11/54 (20%) of cases with weak or negative staining ($P$=0.001). No significant correlation between PD-ECGF/TP and vascular grade was found in patients with adenocarcinomas in which overexpression of PD-ECGF was seen in 4/11 (36%) patients with high vascular grade tumours and 8/27 (28%) with low/medium vascular grade tumours ($P$=0.1). Figure 1A and B show sequential sections of a squamous cell tumour with high PD-ECGF/TP expression and high vascular grade.

### PD-ECGF/TP correlation with other tumour variables

The relationship of PD-ECGF/TP expression with tumour-related parameters is shown in Table 1. Positive expression was observed more frequently in T2-staged cases compared with T1 ($P$=0.04). Twenty out of 62 (32%) T2 cases showed a positive reactivity *vs* 7/45 (15%) T1 cases. No correlation was found between PD-ECGF/TP positive staining and nodal status, grade or histology, or Ki67, EGFR or p53 expression.

**Figure 1** A case of squamous cell lung cancer with positive PD-ECGF expression (**A**) and high vascular grade (**B**)

### Overall survival and PD-ECGF expression

Univariate analysis of survival showed that vascular grade ($P$= 0.0004), nodal status ($P$=0.0015) and T stage ($P$=0.02) were the most significant prognostic variables. PD-ECGF/TP expression did not prove to be of prognostic significance when analysing all NSCLC patients ($P$=0.09) or when analysing those patients with squamous cell tumours ($P$=0.23) or adenocarcinomas ($P$=0.31). A multivariate analysis did not show an independent role for any one of these tumour parameters in overall survival. This is probably because of the strong association of angiogenesis with nodal status and PD-ECGF/TP expression, and of PD-ECGF/TP expression with T stage.

Kaplan–Meier survival curves for all patients (Figure 2A), N0-staged (Figure 2B) and N1-staged cases (Figure 2C) are shown in Figure 2. Overall survival was not statistically worse for PD-ECGF/TP-positive cases when all cases were taken into account ($P$ = 0.09; Figure 2A). However, a statistically significant worse survival was confirmed for N0-staged patients with positive PD-ECGF/TP expression ($P$ = 0.04; Figure 2B).

In Figure 3, Kaplan–Meier survival curves are shown for patients stratified for vascular grade and PD-ECGF/TP expression. High vascular grade defined a worse prognosis in cases with negative/weak PD-ECGF/TP expression ($P$ = 0.0001). In cases with

© Cancer Research Campaign 1997

**Figure 2** Kaplan–Meier survival curves with respect to PD-ECGF expression for all (**A**), NO-staged (**B**) and N1-staged cases (**C**). –, Negative PD-ECGF expression; w, weak expression; +, positive/high expression. There was a significant difference in outcome between combined negative/weak (a/b) and high PD-ECGF expression tumours (*P*=0.04) in N0 disease positive PD-ECGF/TP, high or low vascular grade did not show a significant survival difference.

### DISCUSSION

PD-ECGF/TP is a protein with a wide range of activities, including stimulation of DNA synthesis (Sotos et al, 1994), angiogenesis (Moghaddam et al, 1992; Haraguchi et al, 1993) and endothelial cell migration (Moghaddam et al, 1995). High levels of PD-ECGF/TP are observed in a variety of human tumours (Kono et al, 1984; Vertongen et al, 1984; Yoshimura et al, 1990), and a higher level of serum PD-ECGF/TP is observed in cancer

**Figure 3** Kaplan–Meier survival curves stratified for vascular grade and PD-ECGF expression. VG L/M, low/medium vascular grade; VG H, high vascular grade; –/w, combined negative and weak PD-ECGF expression tumours; +, high PD-ECGF expression tumours patients compared with healthy control patients (Pauly et al, 1977). Apart from neoplastic disorders, angiogenesis plays a critical role in the pathogenesis of a number of benign diseases, such as osteo- and rheumatoid arthritis (Brown et al, 1988; Koch et al, 1994). Recent evidence suggests that PD-ECGF/TP plays a role in the pathogenesis of rheumatoid arthritis through stimulation of angiogenesis (Takeuchi et al, 1994).

The correlation of PD-ECGF/TP expression in human tumours with prognostic variables has been evaluated in other tumour types. Toi et al (1995) showed a statistically significant correlation of PD-ECGF/TP expression with high vascular grade in breast cancer. In contrast to these data, Fox et al (1996) failed to show any relation of PD-ECGF/TP expression with vascular grade in breast cancer. Moreover, a strong inverse correlation of PD-ECGF/TP expression with T stage and grade was observed. In a study on gastric cancer, Maeda et al (1995) found no correlation between PD-ECGF/TP immunoreactivity and histology, depth of tumour invasion or nodal involvement.

Heldin et al (1993) studied the PD-ECGF/TP expression in non-small-cell lung cancer lines. An association was found between positive expression and well-differentiated cell lines. However, we demonstrated that a similar relationship was not the case in surgical specimens from patients with operable non-small-cell lung cancer. PD-ECGF/TP expression was not related to tumour grade, nodal status or histology or Ki67, EGFR or p53 expression. We observed a statistically significant direct correlation of PD-ECGF/TP expression with T stage ($P = 0.04$). Positive expression of PD-ECGF/TP was associated with high vascular grade in non-small-cell lung carcinomas ($P = 0.01$).

In a previous study, we observed that PD-ECGF/TP expression in breast cancer was associated with better prognosis in patients with nodal involvement (Fox et al, 1996), which is in contrast with our findings in non-small-cell lung cancer. This discrepancy may be explained by the fact that node-positive breast cancer patients receive adjuvant chemotherapy including 5-fluorouracil. PD-ECGF/TP is one of the enzymes involved in the transformation of 5-fluorouracil to metabolites that bind to thymidylate synthase, resulting in defective DNA synthesis and repair (Sotos et al, 1994). Cells transfected with PD-ECGF/TP have an increased sensitivity to pyrimidine antimetabolites (Haraguchi et al, 1993; Patterson et al, 1995). Whether 5-fluorouracil-based adjuvant chemotherapy would be effective in PD-ECGF/TP-expressing

© Cancer Research Campaign 1997

non-small-cell lung cancer is unknown. Nonetheless, it may be worth assessing the anti-tumour activity of 5-fluorouracil and 5-fluorouracil prodrugs in PD-ECGF/TP-positive non-small-cell lung cancers.

In conclusion, the results of this study suggest that PD-ECGF/TP has a role in the pathogenesis of non-small-cell lung cancer. High expression of PD-ECGF/TP in non-small-cell lung cancer cells appears to define a more aggressive tumour phenotype with a poorer prognosis, especially in cases without nodal involvement. This is only partially attributable to the positive correlation of PD-ECGF/TP expression with high tumoral angiogenesis. Although the statistically significant association between high vascular grade and positive PD-ECGF/TP expression cannot prove a causative correlation, our results further support previous in vitro observations on the angiogenic role of PD-ECGF/TP in malignant disease. Vascular grade, together with PD-ECGF/TP expression, could be of importance in defining groups of patients with poor prognosis that would benefit from pyrimidine anti-metabolite-based chemotherapy. These observations may be of clinical value in designing randomized trials on adjuvant chemotherapy in operable non-small-cell lung cancer.

### ACKNOWLEDGEMENT

This study was supported by the Imperial Cancer Research Fund.

### REFERENCES

Altman DG (1991) *Practical Statistics for Medical Research*. Chapman & Hall: London.

Barton GJ, Ponting CP, Spraggon G, Finnis C and Sleep D (1992) Human platelet-derived endothelial cell growth factor is homologous to *Escherichia coli* thymidine phosphorylase. *Protein Sci* **1**: 688–690

Brown RA and Weiss JB (1988) Neovascularisation and its role in the ostoarthritic process. *Ann Rheum Dis* **47**: 881–885

Cox DR (1972) Regression models and life tables (with discussion). *J R Stat Soc* **34**: 187–220

Eda H, Fujimoto K, Watanabe S, Ura M, Hino A, Tanaka Y, Wada K and Ishitsuka H (1993) Cytokines induce thymidine phosphorylase expression in tumor cells and make them more susceptible to 5′-deoxy-5-fluorouridine. *Cancer Chemother Pharmacol* **32**: 333–338

Fox SB, Moghaddam A, Westwood M, Turley H, Bicknell R, Gatter KC and Harris AL (1995) Platelet derived endothelial cell growth factor/thymidine phosphorylase expression in normal tissues: an immunohistochemical study. *J Pathol* **176**: 183–190

Fox SB, Westwood M, Moghaddam A, Comley M, Turley H, Whitehouse RM, Bicknell R, Gatter KC and Harris AL (1996) The angiogenic factor platelet-derived endothelial cell growth factor/thymidine phosphorylase is up-regulated in breast cancer epithelium and endothelium. *Br J Cancer* **73**: 275–280

Furukawa T, Yoshimura A, Sumizawa T, Haraguchi M, Akiyama S, Fukui K, Ishizawa M and Yamada Y (1992) Angiogenic factor (letter). *Nature* **356**: 668

Giatromanolaki A, Koukourakis M, O'Byrne K, Fox S, Whitehouse R, Talbot DC, Harris AL and Gatter KC (1996) Prognostic value of angiogenesis in operable non small cell lung cancer. *J Pathol* **179**: 80–88

Haraguchi M, Furukawa T, Sumizawa T and Akiyama S (1993) Sensitivity of human KB cells expressing platelet-derived endothelial cell growth factor to pyrimidine antimetabolites. *Cancer Res* **53**: 5680–5682

Haraguchi M, Miyadera K, Uemura K, Sumizawa T, Furukawa T, Yamada K and Akiyama S-I (1994) Angiogenic activity of enzymes. *Nature* **368**: 198

Heldin NE, Usuki K, Bergh J, Westermark B and Heldin CH (1993) Differential expression of platelet-derived endothelial cell growth factor/thymidine phosphorylase in human lung carcinoma cell lines. *Br J Cancer* **68**: 708–711

Horak ER, Leek R, Klenk N, Lejeune S, Smith K, Stuart N, Greenall M, Stepniewska K and Harris AL (1992) Angiogenesis, assessed by platelet/endothelial cell adhesion molecule antibodies, as indicator of node metastases and survival in breast cancer. *Lancet* **340**: 1120–1124

Ishikawa F, Miyazono K, Hellman U, Dexler H, Wernstedt C, Hagiwara K, Usuki K, Takaku F, Risau W and Heldin CH (1989) Identification of angiogenic activity and the cloning and expression of platelet-derived endothelial cell growth factor. *Nature* **338**: 557–562

Kaplan E and Meier P (1958) Non-parametric estimation from incomplete observations. *J Am Stat Assoc* **53**: 457–481

Koch AE, Harlow LA, Haines GK, Amento, EP, Unemori EN, Wong WL, Pope, RM and Ferrara N (1994) Vascular endothelial growth factor. A cytokine modulating endothelial function in rheumatoid arthritis. *J Immunol* **152**: 4149–4156

Kono A, Hara Y, Sugata S, Matsushima Y and Ueda T (1984) Substrate specificity of a thymidine phosphorylase in human liver tumor. *Chem Pharm Bull* **32**: 1919–1921

Mclaren R, Kuzu I, Dunnil M, Harris AL, Lane D and Gatter KC (1992) The relationship of p53 immunostaining to survival in carcinoma of the lung. *Br J Cancer* **66**: 735–738

Maeda K, Chung YS, Ogawa Y, Takatsuka S, Sawada T, Onoda N, Nitta A, Arimoto Y and Sowa M (1995) Malignancy of gastric cancer analyzed by the expression of thymidine phosphorylase. *Gan To Kagaku Ryoho* **22**: 679–682

Mantel N (1966) Evaluation of survival data and two new rank statistics arising in is consideration. *Cancer Chemother Rep* **50**: 163–170

Moghaddam A and Bicknell R (1992) Expression of platelet-derived endothelial cell growth factor in *Escherichia coli* and confirmation of its thymidine phosphorylase activity. *Biochemistry* **31**: 12141–12146

Moghaddam A, Zhang HT, Fan TPD, HU D-E, Lees V, Turley H, Fox SB, Gatter KC, Harris AL and Bicknell R (1995) Thymidine phosphorylase is angiogenic and promotes tumor growth. *Proc Natl Acad Sci USA* **92**: 998–1002

Mountain CF (1986) A new international staging system for lung cancer. *Chest* **89** (suppl.): 225–233

Miyazono K, Okabe T, Urabe A, Takaku F and Heldin CH (1987) Purification and properties of an endothelial cell growth factor from human platelets. *J Biol Chem* **262**: 4098–4103

Parums DV, Cordell JL, Micklem K, Heryet AR, Gatter KC and Mason DY (1990) JC70: a new monoclonal antibody that detects vascular endothelium associated antigen on routinely processed tissue sections. *J Clin Pathol* **43**: 752–757

Patterson A, Zhang H, Moghaddam A, Bicknell R, Talbot D, Stratford J and Harris A (1995) Increased sensitivity to the pro-drug 5′-deoxy-5-fluorouridine and modulation of 5′-fluoro-2′-deoxyuridine sensitivity in MCF-7 cell transfected with thymidine phosphorylase. *Br J Cancer* **72**: 669–675

Pauly JL, Schuller MG, Zelcer AA, Kirss TA, Gore SS and Germain MJ (1977) Identification and comparative analysis of thymidine phosphorylase in the plasma of healthy subjects and cancer patients. *J Natl Cancer Inst* **58**: 1587–1590

Schwartz EL, Baptiste N, O'Connor CJ, Wadler S and Otter BA (1994) Potentiation of the antitumor activity of 5-fluorouracil in colon carcinoma cells by the combination of interferon and deoxyribonucleosides results from complementary effects on thymidine phosphorylase. *Cancer Res* **54**:1472–1478

Sotos GA, Grogan L and Allegra CJ (1994) Preclinical and clinical aspects oF biomodulation of 5-fluorouracil. *Cancer Treat Rev* **20**: 11–49

Takeuchi M, Otsuka T, Matsui N, Asai K, Hirano T, Moriyama A, Isobi I, Eksiogui YZ, Maksukawa K, Kato Tand Tada T (1994) Aberrant production of gliostatin/platelet derived endothelial cell growth factor in rheumatoid synovium. *Arthritis Rheum* **37**: 662–672

Toi M, Hoshina S, Taniguchi T, Yamamoto Y, Ishitsuka H and Tominaga T (1995) Expression of platelet-derived endothelial cell growth factor/thymidine phospshorylase in human breast cancer. *Int J Cancer* **64**: 79–82

Tungekar MF, Gatter KC, Dunnil MS and Mason DY (1991) Ki-67 immunostaining and survival in operable lung cancer. *Histopathology* **19**: 545–550

Usuki K, Saras J, Waltenberger J, Miyazono K, Pierce G Thomason A and Heldin CH (1992) Platelet-derived endothelial cell growth factor has thymidine phosphorylase activity. *Biochem Biophys Res Commun* **184**: 1311–1316

Veale D, Aschroft T, Gibson, GJ and Harris AL (1987) Epidermal growth factor receptors in non small cell lung cancer. *Br J Cancer* **55**: 513–516

Vertongen F, Fondu P, Van Den Heule B, Cauchie C and Mandelbaum IM (1984) Thymidine kinase and thymidine phosphorylase activities in various types of leukemia and lymphoma. *Tumour Biol* **5**: 303–311

Yoshimura A, Kuwazuru Y, Furukawa T, Yoshida H, Yamada K and Akiyama S (1990) Purification and tissue distribution of human thymidine phosphorylase; high expression in lymphocytes, reticulocytes and tumors. *Biochim Biophys Acta* **1034**: 107–113

© Cancer Research Campaign 1997